# A two year clinical, immunological and gut-brain-axis prospective study to predict treatment response to cladribine tablets in relapsing-remitting multiple sclerosis

**DOI:** 10.3389/fimmu.2026.1955097

**Published:** 2026-10-19

**Authors:** Jeske van Pamelen, Chaja M. J. van Ansenwoude, Maria Miguélez Sánchez, Lotte Spijkerbosch, Carla Rodriguez-Mogeda, Lynn van Olst, Susanne M. A. van der Pol, Maarten L. Boon, Marleen J. A. Koel-Simmelink, Tom van Seeters, Birgit I. Lissenberg-Witte, Janet de Beukelaar, Jiske Fermont, Stephan T. F. M. Frequin, Oliver H. H. Gerlach, Guy Laureys, Bob W. van Oosten, Judith van Vliet, Joep Killestein, Charlotte E. Teunissen, Andries E. Budding, Helga E. de Vries, Leo H. Visser

**Affiliations:** 1 Department of Neurology, Elisabeth-TweeSteden Hospital, Tilburg, Netherlands; 2 MS Center Amsterdam, Amsterdam Neuroscience, Amsterdam UMC Location Vrije Universiteit Amsterdam, Amsterdam, Netherlands; 3 Department of Molecular Cell Biology and Immunology, Amsterdam Neuroscience, Amsterdam UMC Location Vrije Universiteit Amsterdam, Amsterdam, Netherlands; 4 inBiome, Amsterdam, Netherlands; 5 Division of Human Nutrition and Health, Wageningen University and Research, Wageningen, Netherlands; 6 Neurochemistry Laboratory, Department of Laboratory Medicine, Amsterdam Neuroscience, Amsterdam UMC Location Vrije Universiteit Amsterdam, Amsterdam, Netherlands; 7 Department of Radiology, Elisabeth-TweeSteden Hospital, Tilburg, Netherlands; 8 Department of Epidemiology and Data Sciences, Amsterdam UMC, Vrije Universiteit Amsterdam, Amsterdam, Netherlands; 9 Department of Neurology, Albert Schweitzer Hospital, Dordrecht, Netherlands; 10 Department of Neurology, Amphia Hospital, Breda, Netherlands; 11 Department of Neurology, St. Antonius Hospital, Nieuwegein, Netherlands; 12 Department of Neurology, Zuyderland Medical Center, Sittard-Geleen, Netherlands; 13 School for Mental Health and Neuroscience, Maastricht University, Maastricht, Netherlands; 14 Department of Neurology, University Hospital Ghent, Ghent, Belgium; 15 Department of Neurology, Jeroen Bosch Hospital, ‘s Hertogenbosch, Netherlands

**Keywords:** 16S-23S rDNA interspace profiling, cladribine tablets, gut-brain-axis, mass cytometry by time-of-flight, microbiota, relapsing-remitting multiple sclerosis, treatment responder

## Abstract

**Introduction:**

In relapsing-remitting multiple sclerosis (RRMS) it is challenging to predict which person will benefit from a certain therapy, such as cladribine tablets (CladT). This study aimed to obtain a better understanding of the interaction between the gut-brain axis and CladT to improve treatment response prediction.

**Methods:**

In this multicenter, prospective, observational study, we included 81 people with RRMS starting CladT. We assessed the clinical, immunological and microbiological profiles at baseline and after 3, 12 and 24 months, and compared results between responders and non-responders, classified based on inflammatory disease activity and disability progression (NEDA-3).

**Results:**

A total of 35 of 81 (47%) participants were responders. We found no differences in immune cell profiles between responders and non-responders. Network analysis revealed more positive correlations between immune cell clusters within the same parent cluster and a stronger negative correlation between CD8^+^ T cells and myeloid cells in responders. Microbiological analyses showed that fecal alpha diversity, both overall and for Bacteroidetes, as well as the relative abundance of the species *Bacteroides caccae* and *Prevotella copri* in fecal samples, are associated with treatment response to CladT.

**Discussion:**

Although we found a trend toward more coordinated immune reorganization in CladT responders, and identified fecal alpha diversity and fecal *Bacteroides caccae* and *Prevotella copri* as associated with treatment response, we could not develop a reliable model to predict treatment response before or shortly after the start of CladT. Our results warrant further investigation in larger cohorts.

## 1 Introduction

Personalized treatment in people with relapsing-remitting multiple sclerosis (RRMS) is an emergent area of interest, as the spectrum of possible immunomodulatory therapies (IMT) has expanded considerably in the last decades. However, it is still challenging to predict if a person will benefit from a certain therapy. A possible approach to better understand individual treatment responses is to examine the interaction between the gut, brain and immune system, commonly referred to as the gut-brain axis or brain-immune-intestine axis.

An interesting IMT to study this gut-brain axis is cladribine, as cladribine has been shown to elicit a local immunological response in the gut in people with refractory celiac disease (1). Cladribine is a synthetic deoxyadenosine analogue, of which the oral form is registered for the treatment of highly active RRMS (2, 3). Cladribine tablets are an immune reconstitution therapy, which means that short-term immunosuppression can induce long-term remission (2, 4). It is administered during two treatment weeks in the first year and two treatment weeks in the second year (4). In a randomized controlled trial, 44% of the treated participants had no evidence of disease activity (NEDA-3) after 96 weeks, which means that these participants did not experience relapses, showed no disability progression and did not have radiological disease activity on magnetic resonance imaging (MRI) (5). Cladribine tablets (CladT) induce a selective depletion of immune cells, mainly of B lymphocytes and to a lesser extent of T lymphocytes (6–11). Cladribine needs deoxycytidine kinase (DCK) to convert to its active form, which impacts DNA synthesis and repair and promotes apoptosis (12). T- and B-cells have the highest deoxycytidine kinase:5’ nucleotidase (DCK:5-NT) ratio compared to other immune cells, which explains the susceptibility of these cells to cladribine (13).

The gut-brain axis has been studied extensively in both animal models of multiple sclerosis (MS) and in people with MS (pwMS) (14, 15). It has been shown that the gut microbiota composition differs between pwMS and healthy controls (14, 15). Moreover, transplantation of gut bacteria from pwMS to germ-free mice exacerbates experimental autoimmune encephalomyelitis (EAE), a mouse model of MS (16, 17). Furthermore, a small number of studies were able to show that the abundance of several bacterial phyla, genera and species correlated with either a lower number of immunomodulatory cells or a higher number of proinflammatory cells, and that the microbiota composition correlated with disease activity (18–22). Lastly, several longitudinal studies have found microbiota changes after IMT, such as dimethyl fumarate and ocrelizumab (23–26).

The oral-brain axis is still an underexplored area, despite the ease of oral sample collection and the degree of consistency between the microbial compositions of oral and fecal swabs (27). A previous study investigated the role of the oral microbiota in pwMS and found some differences on phyla and genera level between pwMS and healthy controls (28).

Data on microbiota changes after treatment with cladribine and the correlation with disease activity are limited to a recently published interim analysis of the current study, which found that several phyla, genera or species with immunomodulatory properties differed (although not significantly) between responders and non-responders (29). However, due to a small sample size and short follow-up of one year, it was not possible to correct for potential confounders and draw definitive conclusions.

Therefore, in this study, we aimed to describe the changes in the gut and oral microbiota and the immunological profile after completed treatment with CladT and examined whether these changes differed between responders and non-responders two years after the first treatment, in a larger cohort. Moreover, our objective was to determine if the gut and oral microbiota at baseline or after three months are a predictor for treatment response to CladT after two years.

## 2 Methods

### 2.1 Population

Data were used from the multicenter, prospective, observational BIA (Brain-Immune-Intestine Axis) study, which recruited participants from eleven outpatient clinics in the Netherlands and Belgium between January 2019 and October 2022 (30). Inclusion criteria were a diagnosis of RRMS, a scheduled start of CladT as per standard of care and age between 18 and 60 years. Exclusion criterium was use of probiotics within one month prior to the planned start of CladT.

### 2.2 Study procedures

Participants were followed during two to two and a half years, with study visits carried out at baseline, after three, twelve and twenty-four months (D0, M3, M12, M24, displayed in **Figure 1**). During each visit, the Expanded Disability Status Scale (EDSS) was assessed and blood, fecal and oral samples were collected. Participants were asked to report the consistency of their feces using the Bristol stool scale and to record their intake for seven days after each visit. As per standard of care, the second treatment cycle was administered once the lymphocyte count had recovered to >0.8x10^9^/L and a brain MRI was performed 12 months after each treatment course. In case of a relapse, an extra visit took place within 14 days from onset of symptoms, to determine the EDSS.

**Figure 1:**
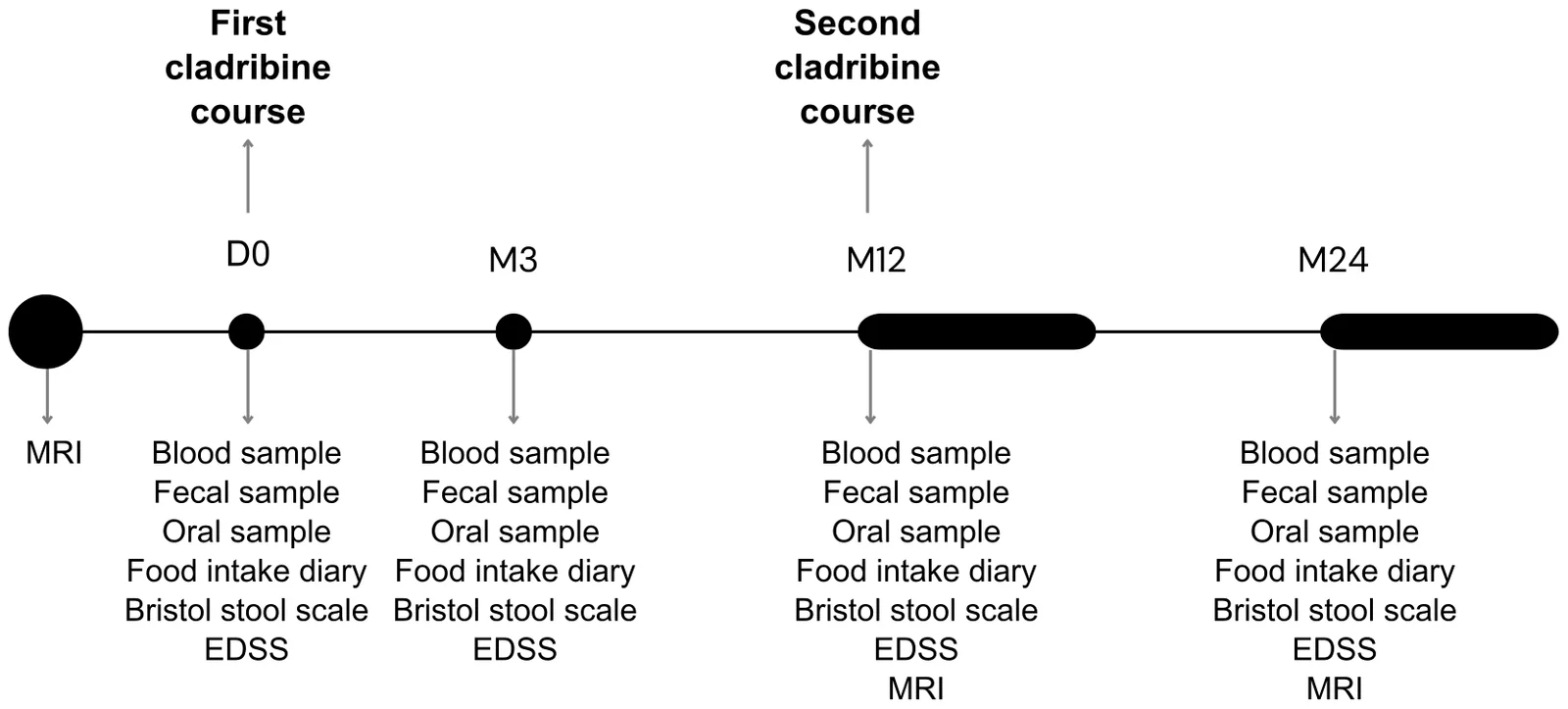
Study timelines. Baseline MRI had to be performed within 3 months before scheduled start of cladribine tablets as per standard of care. D0 (baseline) was performed within one week prior to the start of cladribine tablets. M3 was performed 3 months after D0. M12 timing varied from 12–18 months, as it took place prior to the start of the second course of cladribine, or after 18 months if a second treatment course was not possible (mostly because of lymphopenia). M24 took place 12 months after M12.

### 2.3 Outcome measures

#### 2.3.1 Response status

Response status (‘overall response status’) was determined at M24 and based on the three pillars of ‘no evidence of disease activity’ (NEDA-3): no relapses, no radiological activity and no EDSS progression (31). Radiological activity was assessed by a central radiologist, blinded to clinical and laboratory data, based on brain MRI performed within 3 months before baseline and at 12 and 24 months. According to the conventional NEDA-3 definition, a participant was considered a non-responder if either one of the three pillars was positive for disease activity. The response status could still be determined in case of missing parameters if one of the available parameters classified the participant as a non-responder. Otherwise, the response status was set as ‘missing’. More detailed definitions of a relapse, EDSS progression and radiological activity were published before (29).

As CladT treatment consists of two treatment courses, we decided to additionally analyze the response status between M12 and M24, where relapses, radiological activity and EDSS progression between D0 and M12 were not taken into account (‘year 2 response status’).

#### 2.3.2 Immunological profile

##### 2.3.2.1 Immune cell isolation

Peripheral blood mononuclear cells (PBMCs) were isolated from blood using Vacutained Mononuclear Cell Preparation Tubes with sodium citrate (CPT, BD Biosciences) according to manufacturer’s instructions. PBMCs were divided into 5x10^6^ cells per cryovial and stored at -80 °C for a maximum of three months, after which they were transferred to liquid nitrogen until staining and analysis for mass cytometry by time-of-flight (CyTOF).

##### 2.3.2.2 Generation of a reference sample for CyTOF measurements

Reference samples were generated to control for potential batch effects in signal intensity. These samples were created by isolating PBMCs from buffy coats of two healthy donors (Sanquin Blood Bank, Netherlands) using a standard density gradient centrifugation protocol with Lymphoprep (StemCell Technologies). The PBMCs of one donor were enriched for B cells using the EasySep Human B cell enrichment kit (StemCell Technologies). Half of the PBMCs from the second donor were divided equally and stimulated with IL-1β (10 ng/mL, PeproTech), IL-4 (10 ng/mL, ImmunoTools), LPS (10 ng/mL, Sigma), TGF-β1 (100 ng/mL, Miltenyi), Dexamethasone (100 nM, Sigma), TNF-α (10ng/mL, PeproTech), IFN-γ (10 ng/mL, PeproTech), PolyIC (500 ng/mL, Amersham Pharmacia Biotech), or a mix of PMA (25 ng/mL, Sigma) and ionomycin (100 ng/mL, Sigma) at 37 °C for 12 hours to induce expression of various markers included in the CyTOF panel. The unstimulated and stimulated PBMCs as well as PBMCs enriched for B cells were combined and stored at -70 °C in 5×10^6^ cells/cryovial until staining and analysis.

##### 2.3.2.3 CyTOF staining

PBMCs were stained using the MaxPar Direct Immune Profiling Assay (Standard BioTools) according to manufacturer’s instructions. Two reference samples were included in each batch of samples to stain. After thawing, cells were resuspended in fetal calf serum (FCS, Corning) and washed twice using RPMI medium 1640 (Gibco) supplemented with 10% FCS. All centrifugation steps were performed at 4 °C for 7 min at 500 × g (before fixation) or 800 × g (after fixation). Cells were then incubated in RPMI supplemented with DNase I (1 µg/mL, Merck) for 30 minutes at 37 °C, followed by a wash using Cell Staining Buffer (CSB, Standard BioTools). All incubation steps were carried out on a shaker at room temperature unless specified otherwise. Next, cells were incubated with Human TruStain FcX (1:50 in CSB; Biolegend) for 10 min. Each sample was then barcoded using cadmium-conjugated CD45 barcoding antibodies (Standard BioTools) for 30 min. After two washes with CSB, samples were pooled and stained with the MaxPar Direct Immune Profiling Assay for 30 min. Cells were washed twice with CSB and once with MaxPar PBS (Standard BioTools) before fixation using 1.6% paraformaldehyde (Thermo Fisher) for 15 min. Finally, cells were incubated with 125 nM Cell-ID Intercalator-Ir solution Standard BioTools) for 1 hour. Cells were divided into 5x10^6^ cells per cryovial and stored at -80 °C until acquisition.

##### 2.3.2.4 CyTOF acquisition

Cells were thawed and washed twice with CSB and once with Cell Acquisition Solution Plus (CAS+, Standard BioTools), followed by resuspension in CAS+ at 600 cells/µL. After filtering over a 35 µm cell strainer (Corning), 10% EQ Six Element Calibration Beads (Standard BioTools) was added to each vial. Acquisition was performed using the CyTOF XT™ (Standard BioTools) at 300 to 400 events per second. After start-up of the machine, tuning of the machine was performed. After each sample, the system was washed using MaxPar Washing Solution (Standard BioTools) for 5.5 min at 50 µL/min and rinsed with MaxPar Water (Standard BioTools) and CAS +. Washing time was extended to 8.3 minutes when samples were part of different batches.

##### 2.3.2.5 CyTOF data processing

Acquired data were randomized and normalized to beads according to the default settings in the CyTOF software, followed by file concatenation (Standard BioTools, version 8.1.0.18524). Further processing was performed using OMIQ (Dotmatics, www.omiq.ai). First, cell debris, beads, and doublets were removed using a gating strategy based on markers for DNA, beads, Gaussian parameters, and CD45 (32). To remove moments of aberrant signal intensity during acquisition, the *FlowCut* package in OMIQ was used (33). Debarcoding of samples was performed in Rstudio (version 4.3.2) using *CATALYST* (34). Dead cells were excluded in OMIQ based on negative Cell-ID Cisplatin signal. Samples with viability below 50% and live cell count below 10,000 were excluded, and samples of one participant were excluded due to comorbid diagnosis of COPD, which can give a bias in the amount of CD8^+^ Effector Memory T cells. Mean cell viability of included samples was 91.0% ± 7.1% (Standard Deviation). Immune cell clusters were identified using a gating strategy based on canonical lineage markers (**Supplementary Figure 1**). Clusters were visualized using Uniform Manifold Approximation and Projection (UMAP) based on 10,000 cells per sample (35).

#### 2.3.3 Microbiological profile

Fecal and oral samples were collected in 1 mL eNat^®^ buffer (608CS01R, Copan). To determine the composition of the gut and oral microbiota, we used the interspace-profiling (IS-pro) technique (Inbiome), which identifies bacteria based on specific-length polymorphisms in the 16S-23S rDNA interspace (IS) region, combined with phylum-specific sequence length polymorphisms in the 16S rDNA (36). IS-pro provides taxonomic profiling at partial species-level resolution, rather than direct sequencing of microbial genes. Data was preprocessed and species calling was done with the software suite antoni (Inbiome) and further analyzed with the Spotfire software package (TIBCO). For the analysis, we used relative abundance of phyla and species, and we calculated the alpha diversity (using the Shannon Diversity index, a measure that reflects both species richness and evenness within a sample (37)) and the *Firmicutes/Bacteroidetes* (F/B) ratio. More detailed data on the performed analysis can be found in a previous publication (29).

#### 2.3.4 Food intake

After each study visit, participants were asked to complete a digital food and beverage diary for seven consecutive days. The diary aimed to capture habitual intake patterns, including specific details about all consumed foods and beverages, in order to analyze changes in dietary patterns in relation to microbiota composition and treatment response. The information from the diaries was analyzed and integrated into food categories, with special focus on dietary patterns with known associations to gut microbiota composition, such as dietary fiber and high-fermented food products (38, 39). Dietary fiber is fermented by gut bacteria into short-chain fatty acids (SCFAs) such as butyrate, propionate, and acetate, which are known for their anti-inflammatory effect (38). High-fermented food products often contain live microorganisms, which have been shown to exert anti-inflammatory effects by modulation of cytokine release and improvement of the gut barrier function (39). Therefore, variation in fiber and high-fermented food intake among participants could possibly confound or explain observed differences between response groups.

To capture dietary fiber intake, we assessed the diaries using the 18-item FiberScreen (version 2), a validated short food frequency questionnaire designed to estimate daily fiber intake in adults, developed and validated by Wageningen Food & Biobased Research (40). The intake of high-fermented food products (such as yogurt and cottage cheese), was extracted from the diaries and calculated as an average number of servings per day.

### 2.4 Statistical analysis

A sample size calculation was performed and resulted in a number of 80 participants that had to be included to obtain sufficient statistical power for a reliable prediction of response status. A more detailed explanation can be found in the previously published research protocol (30).

Statistical analyses were carried out with RStudio version 4.4.3 (immunological profile), GraphPad Prism version 10 (immunological profile and dietary data), Spotfire version 10.6.0 and Phyton version 3.10 (both for microbiological profile as a predictor for response status), and SPSS version 29 (for all other analysis). *P*-values were adjusted for multiple testing using the false discovery rate of Benjamini and Yekutieli (BY) for the linear mixed-effects model (LMM) with immunological measures, and of Benjamini and Hochberg for all other analyses (41, 42).

#### 2.4.1 Baseline characteristics

Baseline characteristics are presented as absolute and relative frequencies for categorical variables and median (interquartile range, IQR) for continuous data. Differences in baseline characteristics between responders and non-responders were investigated using a *t*-test, Mann-Whitney U test or Fisher’s exact test where appropriate.

#### 2.4.2 Immunological profile

The immunological subsets are displayed as mean percentages of the total amount of CD45^+^ cells. The distributions of proportional data were normalized by arcsine square root transformation. An LMM was then fitted with the proportion of cells per cluster as the outcome variable, the visit, response status, and their interaction as fixed effects, age and sex as covariates, and a random intercept for each participant. Model assumptions were tested with residual diagnostics including Q-Q plots to assess normality and residuals versus fitted plots for homoscedasticity. The interaction term was assessed using a Type III ANOVA. *P*-values for *post hoc* comparisons between timepoints within either the responder or non-responder groups were obtained using estimated marginal means in the *emmeans* package.

Associations between cluster proportions and clinical data were performed using Spearman correlation for continuous variables, Mann-Whitney U test for binomial variables, and Kruskal-Wallis test followed by a *post hoc* Dunn’s test for variables with more than two groups.

Besides looking only at proportional changes of immune cells, we also performed a network analysis to study the correlation between immune cell clusters. To evaluate whether overall immune network organization differed between responders and non-responders, Spearman correlation matrices were generated separately for each group at each timepoint using all measured immune cell populations. Global differences between correlation matrices were quantified using the Frobenius distance between matrices. Statistical significance was assessed using a permutation test (2.000 permutations), in which responder/non-responder labels were randomly reassigned while preserving group sizes. For each permutation, correlation matrices and their Frobenius distance were recalculated, generating a null distribution from which *p*-values were derived. For visualization purposes, correlation networks were generated using pairwise Spearman correlations. Network graphs were generated with the *igraph* and *ggraph* packages, with nodes representing clusters sized according to mean cluster proportion. Correlations between clusters were analyzed using pairwise Spearman rank correlations per visit within either the responder or non-responder group using the corr.test function from the *psych* package. Only correlations with an absolute correlation coefficient <-0.5 or >0.5 and a BY-adjusted *p*-value <0.05 were visualized.

#### 2.4.3 Microbiological profile

The microbiological measures are displayed as relative abundances over the total bacterial material present in each sample. These data were also analyzed using an LMM, where time was used as fixed effect and participant as random intercept. To analyze differences between responders and non-responders in the LMM, response status was added as main fixed effect and as interaction term to fixed time effect. To compare responders and non-responders at each individual timepoint, we derived the estimated marginal means for each group at each timepoint, along with pairwise group comparisons at each timepoint based on these model-derived estimates.

For the analysis of changes between time points, we only used the samples which had a correct timing of D0 and M3, and a timing of their M12 visit 12 months after the first treatment course and of their M24 visit both 12 months after the second treatment course and 24 months after the first treatment course, as otherwise, there could be a large variation of findings. Potential confounders (baseline characteristics and Bristol stool scale) were added to the LMM to check if they influenced the microbiological measures. The confounder ‘immunomodulatory therapy (IMT) used in the last 6 months’ was divided into three categories (platform, high efficacy and no IMT). The confounder Bristol stool scale was divided into two categories (0–3 and 4-7).

The assumption of a normal distribution of the residuals was met for phyla and diversity measures, but not for the species and the F/B ratio. The F/B ratio was logarithmic transformed, which led to a normal distribution of the residuals, but no normal distribution could be reached for the LMM of the bacterial species. Therefore, we performed a sensitivity analysis, where we excluded the samples with extreme outliers (>3x IQR) per measure to check if conclusions remain valid. This was the case for all the conclusions presented in this manuscript. In cases where the final Hessian matrix was not positive definite and/or iteration was terminated but convergence had not been achieved, a different LMM without a random intercept for participant was used to model the dependency in the repeated measurements.

To determine if the gut and oral microbiota at baseline or after three months are a predictor for treatment response to CladT after two years, several approaches were used. First, unsupervised clustering was used as an approach for microbiota profile evaluation. Clusters were visualized using UMAP. For each sample, a feature vector was constructed from the raw bacterial profiles (fragment length vs fragment intensity). Pairwise sample distances were computed using cosine distance and used as the input for UMAP. Additionally, average IS-pro profiles per patient across all time points were clustered using Principal Coordinates Analysis (PCoA), with cosine distance as distance metric, and Permutational Multivariate Analysis of Variance (PERMANOVA) to assess the statistical differences between the groups.

Second, a preliminary supervised machine learning approach was applied to explore the prediction effect of the microbiology profiles. The selected approach was a Random Forest classifier (n_estimators=100, class_weight=‘balanced’), which used as input variables the bacterial abundance and alpha diversity of baseline and M3 oral and fecal samples. Due to sample size restrictions, we employed Group 5-Fold cross-validation (20/80 test-train split per fold) stratified by participant, to prevent data leakage from sample similarities between baseline and M3. The target variable for prediction used was the ‘overall response status’, and performance is reported using accuracy overall folds and its standard deviation. Feature importances are reported *post hoc* for biological interpretation.

The last strategy used was a supervised classification approach better suited for smaller sample sizes, adaptive group-regularized logistic ridge regression (AGRR) (43). This approach is well suited for high dimensional data with low sample numbers and was applied as previously described in the published protocol with some adaptations (30). The model’s input consisted of the relative abundances and Shannon diversity of both fecal and oral samples at baseline and the species’ phylum (FAFV, Bacteroidetes and Proteobacteria) and sample origin (Fecal and Oral) were used as co-data. The target variable for prediction was the ‘overall response status’ as well. The model was trained using a leave-one-out cross-validation and the results were reported as area under the curve (AUC), accuracy, positive predictive value (PPV), negative predictive value (NPV), sensitivity and specificity (95% confidence intervals). Given the importance of stringent feature selection in diagnostic assay development, we evaluate classifiers across varying feature set sizes (all features without co-data, all features with co-data and top 25 features with co-data). Over each train iteration, *post-hoc* feature selection via an L1 penalty (β-value) was applied, effectively yielding a group-weighted elastic net that accommodates orthogonal effects. Parameter selection was iteratively performed within each training fold.

#### 2.4.4 Association between immunological and microbiological profile

To investigate the association between the immunological and microbiological profile, and to investigate if the interaction differs between responders and non-responders, we ran an LMM with the immunological measures as dependent variable and the microbiological measures (alpha diversity indices, phylum-level relative abundances, and the F/B ratio) as independent variable, the visit, response status, and their interaction as fixed effects, age and sex as covariates, and a random intercept for each participant.

#### 2.4.5 Food intake

Food diary data were excluded from analysis if more than four days of a given 7-day diary were incomplete or missing. Differences in dietary intake between groups were analyzed using independent samples *t-*test or Mann-Whitney U test where appropriate. To correlate dietary intake to microbiological measures, Pearson’s or Spearman’s correlation was used, depending on the distribution of the data. If there were no differences between time points, the average of all time points was used.

## 3 Results

### 3.1 Baseline characteristics

Eighty-one participants were included in the BIA study. The median age of the participants was 41 years and 72% were female. The median disease duration was seven years and the median EDSS at enrollment was 2.0. Fifty-four participants (67%) started CladT because of suffering from disease activity while on other IMT (79% platform therapies, 17% highly effective therapies). Baseline characteristics are summarized in **Table 1**.

**Table 1:** Baseline characteristics.

Characteristics	All participants (N = 81)
Age, years, median (IQR)	41 (31.5-50.0)
Female, n (%)	58 (71.6)
Body mass index, median (IQR)	25.2 (22.4-28.7)
Disease duration, years, median (IQR)	7 (2.0-11.5)
Documented relapses in past 2 years, median (IQR)	1 (0-1)
IMT used in last 6 months, n (%)	
(Peg-)Interferon β	7 (8.6)
Glatiramer acetate	8 (9.9)
Dimethyl fumarate	22 (27.2)
Teriflunomide	19 (23.5)
Fingolimod	8 (9.9)
Natalizumab	2 (2.5)
Ocrelizumab	2 (2.5)
Immunoglobulins	4 (5.0)
None	9 (11.1)
Reason for switch to cladribine tablets, n (%)	
Newly diagnosed active RRMS	9 (11.1)
Disease activity	54 (66.7)
Adverse events	18 (22.2)
Use of systemic corticosteroids in previous month, n (%)	4 (4.9)
Use of antibiotics in previous month, n (%)	9 (11.1)
Smoking, n (%)	
Yes	11 (13.6)
Past	29 (35.8)
Never	41 (50.6)
EDSS at enrollment, median (IQR)	2.0 (1.5-3.5)

EDSS, expanded disability status scale; IMT, immunomodulatory treatment; IQR, interquartile range; RRMS, relapsing-remitting multiple sclerosis.

### 3.2 Response status

Seventy-four participants had sufficient data to determine the ‘overall response status’ (**Supplementary Table 1**).

Baseline MRI was performed a median of 48 days before start of treatment (IQR 21–85 days, range -11–206 days). The M12 MRI followed a median of 392 days after the baseline MRI (IQR 349–439 days, range 193–610 days), and the M24 MRI followed a median of 376 days after M12 MRI (IQR 349–433 days, range 135–630 days). These intervals did not differ between responders and non-responders.

The total number of participants who were considered a responder at M24 was 35 (47%). Of the non-responders, 24 participants (62%) only had disease activity in the first treatment year. Disease activity consisted of relapses in 5 participants (13%), new or growing T2 or gadolinium enhancing lesions on MRI in 27 participants (69%) and of disability progression in 9 participants (23%). Between responders and non-responders, baseline characteristics did not differ significantly, except for a higher BMI in responders (26.7 vs 23.3 in non-responders, *p* = 0.04, **Supplementary Table 2**).

For the ‘year 2 response status’, 71 participants had sufficient data available. Based on the ‘year 2 response status’ definition, 57 (80%) were a responder and 14 (20%) were a non-responders (**Supplementary Table 1**).

### 3.3 Immunological profile

The number of available and correctly timed PBMC samples in both responders and non-responders is displayed in **Figure 2**.

**Figure 2:**
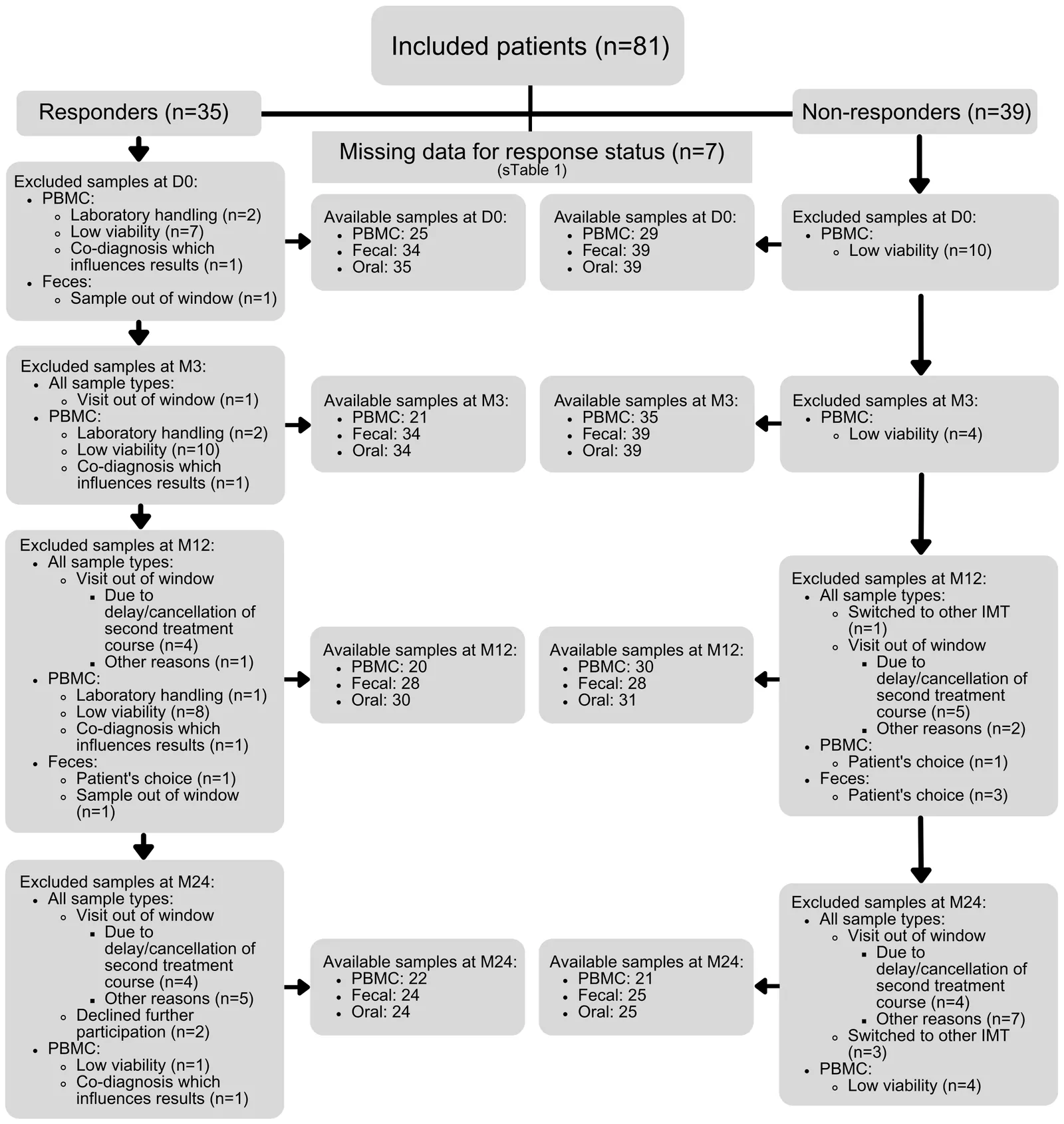
Flowchart of included samples for analysis of changes between time points. IMT, immunomodulatory treatment; PBMC, peripheral mononuclear blood cell; fecal, fecal samples; oral, oral samples; **sTable 1**, **Supplementary Table 1**.

#### 3.3.1 Changes between time points of the immunologic profile

Manual gating of immune cells based on lineage markers resulted in the identification of 21 clusters (**Figures 3A, B**; **Supplementary Figure 1**). We separately analyzed changes between time points in responders and non-responders, and compared the changes between both groups. For each immune cell cluster, there were no significant differences in how the proportions of these clusters out of total immune cells changed between time points for both overall and year 2 response status (**Supplementary Table 3**). However, both responders and non-responders showed strong changes in proportions of various immune cell clusters between time points (**Figures 3C, D**; **Supplementary Figure 2**; **Supplementary Table 3**).

**Figure 3:**
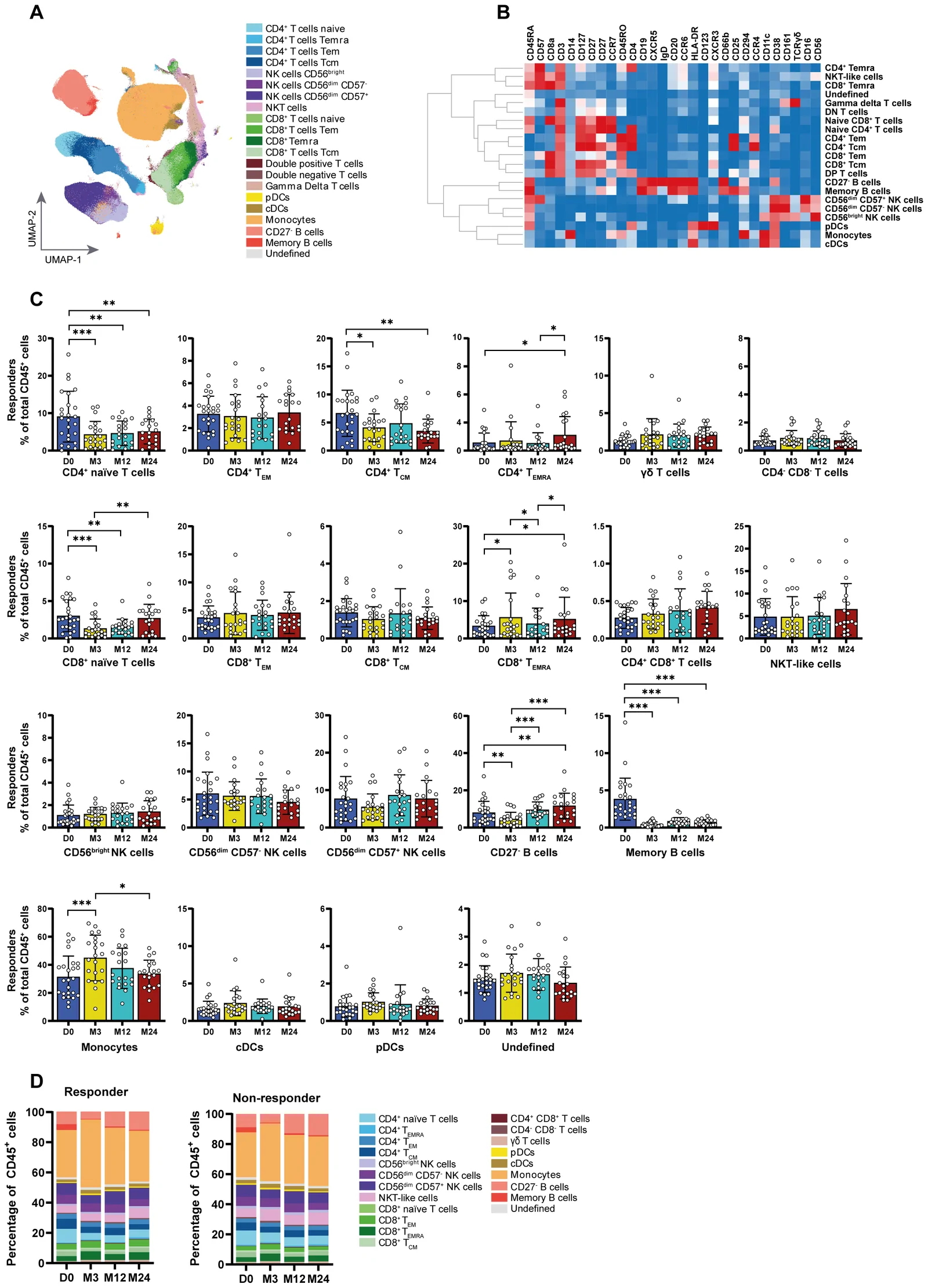
Immune cell population composition is altered by cladribine treatment. **(A)** UMAP overlay plot showing the immune cell clusters identified following manual gating (**Supplementary Figure 1**). **(B)** Heatmap showing the median scaled intensities of markers across the identified clusters. **(C)** Percentages of identified clusters out of the total CD45^+^ cells between time points in responders. Columns and error bars represent mean ± SD, each dot represents an individual donor. Changes in proportions were analyzed using a linear mixed-effects model adjusting for age and sex and including a random intercept for participant. **(D)** Proportional stacked bar graph showing the percentages of clusters out of total CD45^+^ cells between time points in responders and non-responders. Columns depict the mean of all donors. NKT-like cells, natural killer-T like cells; γδ T cells, gamma-delta T cells; T_EMRA_, effector memory cells re-expressing CD45RA; T_CM_, central memory T cells; T_EM_, effector memory T cells; NK cells, natural killer cells; pDCs, plasmacytoid dendritic cells; cDCs, conventional dendritic cells. * p< 0.05 **p<0.01 *** p<0.001.

In the CD4^+^ T cell clusters, we found a significant decrease in CD4^+^ naïve T cells at all time points compared to baseline in both response groups. Furthermore, the proportion of CD4^+^ central memory T cells (T_CM_) was decreased at M3 and M24 compared to baseline, which was only significant in the responders. Another change that was only observed in responders was an increase of CD4^+^ effector memory cells re-expressing CD45RA T cells (T_EMRA_) at M24 compared to D0 and M12. In the non-responders, CD4^-^ CD8^-^ (double negative) T cells were decreased at M24 compared to M3.

In the CD8^+^ T cell clusters, the CD8^+^ naïve T cell proportion declined significantly from D0 to M3 and M12, but not to M24, where their proportion rose again relative to M3. CD8^+^ T_EMRA_ cells increased significantly from baseline to M3 and declined from M3 to M12 in both groups. In responders, CD8^+^ T_EMRA_ cells increased again from M12 to M24, where M24 levels were also significantly higher than baseline levels.

In the B cell clusters, the proportion of both CD27^-^ B cells - the majority of B cells -, and memory B cells declined significantly from baseline to M3 in both groups. From M3 to M12 and M24, the proportion of CD27^-^ B cells recovered, and even increased compared to baseline. In contrast, the proportion of memory B cells did not recover at any time point compared to baseline, although in the non-responder group, it increased from M3 to M12.

Within the myeloid compartment, mostly the proportion of monocytes was affected, with an increase at M3 compared to baseline in both groups, which decreased again to M24 in the responders. In the non-responders, there was a decrease of plasmacytoid dendritic cells from M3 to M24.

Finally, the proportion of natural killer T-cell like cells only changed significantly in non-responders, in which it increased from D0 to M3. No significant changes in natural killer cell populations were observed.

#### 3.3.2 Correlation between immune cell proportions

Besides studying proportional changes of immune cells, we also performed a network analysis to identify correlations between immune cell clusters (**Figure 4**). We found no significant differences between the correlation structure from responders and non-responders, although before correction for multiple testing there was a trend at M3 (*p* = 0.08, adjusted *p* = 0.3). This difference was mainly based on a stronger negative correlation between members of the CD8^+^ cluster and members of the myeloid cell cluster in responders. Although not significantly different, at each time point more positive correlations were present between the different T cell clusters in responders than in non-responders. Positive correlations were mostly identified between clusters of a similar parent cluster, whereas negative correlations were mostly found between immune cells of different parent clusters.

**Figure 4:**
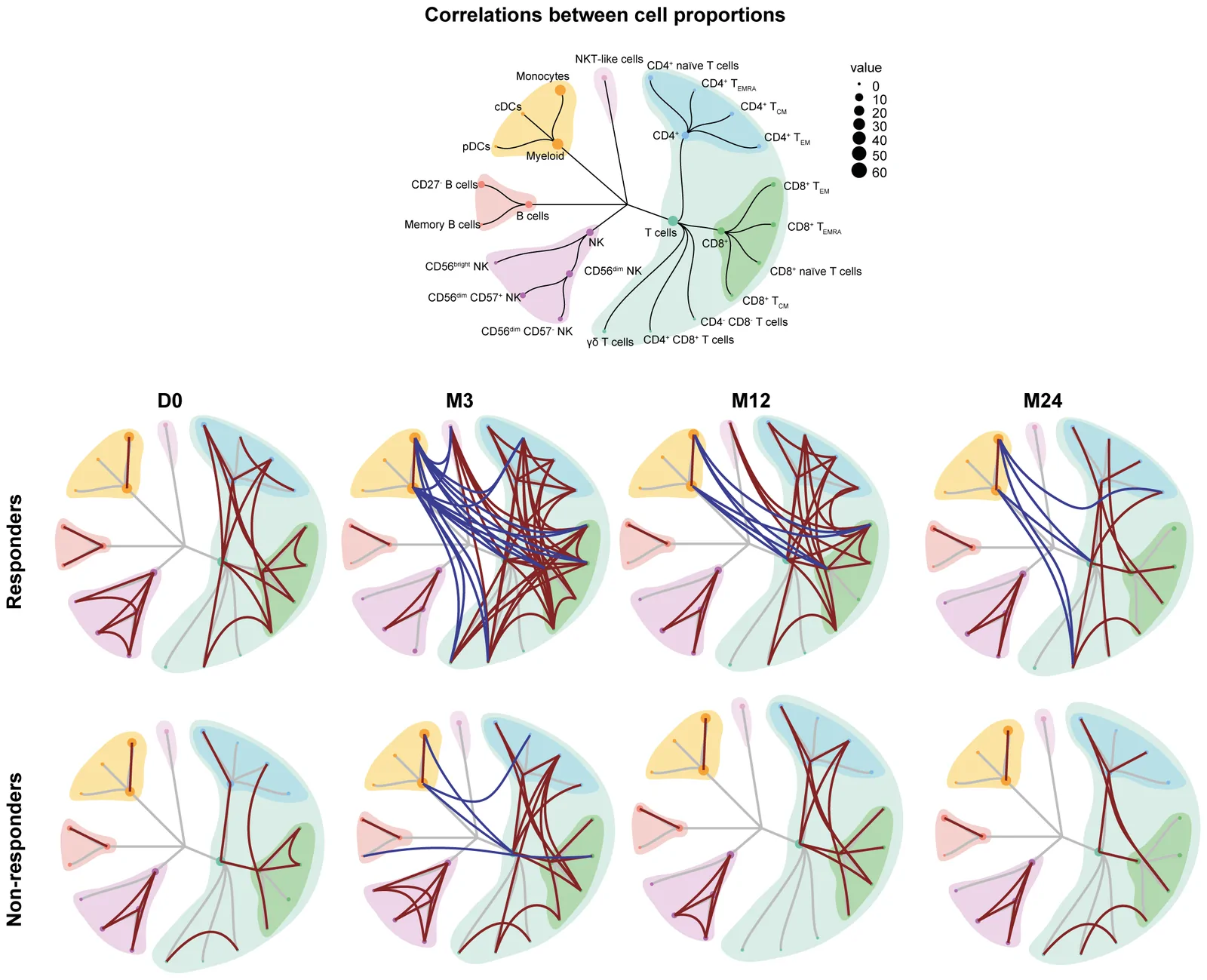
Correlation networks of immune cell clusters in responders and non-responders. Correlations were calculated using spearman correlation, nodes visualize immune cell clusters, red lines represent positive correlations, blue lines represent negative correlations. Only significant correlations with a correlation coefficient above 0.5 or below -0.5 are included. Node size depicts relative cell abundance, color of nodes indicates immune cell parent cluster. NKT-like cells, natural killer-T like cells; γδ T cells, gamma-delta T cells; T_EMRA_, effector memory cells re-expressing CD45RA; T_CM_, central memory T cells; T_EM_, effector memory T cells; NK cells, natural killer cells; pDCs, plasmacytoid dendritic cells; cDCs, conventional dendritic cells.

### 3.4 Microbiological profile

The number of available and correctly timed fecal and oral samples in both responders and non-responders is displayed in **Figure 2**.

#### 3.4.1 Changes between time points

We separately analyzed changes between time points in responders and non-responders (‘overall response status’). No significant changes between time points were found for alpha diversity, F/B ratio, phyla and species in both fecal and oral samples. Between responders and non-responders (both ‘overall response status’ and ‘year 2 response status’), there were no significant differences in changes between time points for alpha diversity, F/B ratio, phyla and species in both fecal and oral samples (**Supplementary Figure 3**). Similarly, pairwise comparisons of estimated marginal means showed no significant differences responders and non-responders at any timepoint.

Several potential confounders (age, sex, BMI, smoke status, disease duration, documented relapses in past two years, use of IMT in the past six months, use of systemic corticosteroids or antibiotics in previous month, baseline EDSS and Bristol stool scale) were added to the LMM. None of them significantly influenced the changes between time points, although smoking status and the Bristol stool scale significantly influenced the results of some parameters themselves (see **Table 2**).

**Table 2:** Significant effects of confounders on microbiological parameters.

Confounders	Microbiological parameters			
	Oral - alpha diversity -proteobacteria	Oral - relative abundance -bacteroidetes	Oral - relative abundance -proteobacteria	Fecal - alpha diversity - bacteroidetes
Smoke status	0.27 ↑ in non-smokers (*p* = 0.005)	5% ↓ in non-smokers (*p* = 0.003)	6% ↑ in non-smokers (*p* < 0.001)	–
Bristol stool scale	–	–	–	0.8 ↑ in grades 0–3 compared to grades 4-7 (*p* < 0.001)

↑ going up, ↓ going down

#### 3.4.2 Predicting response status using microbiological measures

Three different approaches were used to determine if the gut and oral microbiota at baseline or after three months were a predictor for treatment response to CladT after two years.

##### 3.4.2.1 Unsupervised clustering

The unsupervised clustering of the microbiota measures, by generating a UMAP, based on cosine-correlations of paired microbiota profiles, did not reveal a clear distinction between responders and non-responders. Also, the PCoA analysis of the samples grouped by patient did not reveal significant clustering for both the oral and fecal samples (**Supplementary Figure 4**; **Supplementary Table 4**).

##### 3.4.2.2 Random forest model (preliminary machine learning)

To assess whether microbiota composition could discriminate between responders and non-responders, we applied a Random Forest classifier. Each of the 293 fecal and oral samples available were aggregated by patient and timepoint, leaving a total of 148 instances (74 participants x two timepoints each) with 170 informative parameters available for test and training. The best performance of the model (accuracy:0.61, standard deviation:0.05) was obtained including fecal and oral samples from D0 and M3. Given the risk of overfitting and lack of robustness in a small dataset, we did not perform additional hyperparameter tuning or feature selection. Instead, we opted for an explorative approach, highlighting the features that contributed most to each response status. The features with the highest contribution in the model classification were those from the fecal alpha diversity index (**Figure 5**). Generally, low diversity values were associated with non-responders.

**Figure 5:**
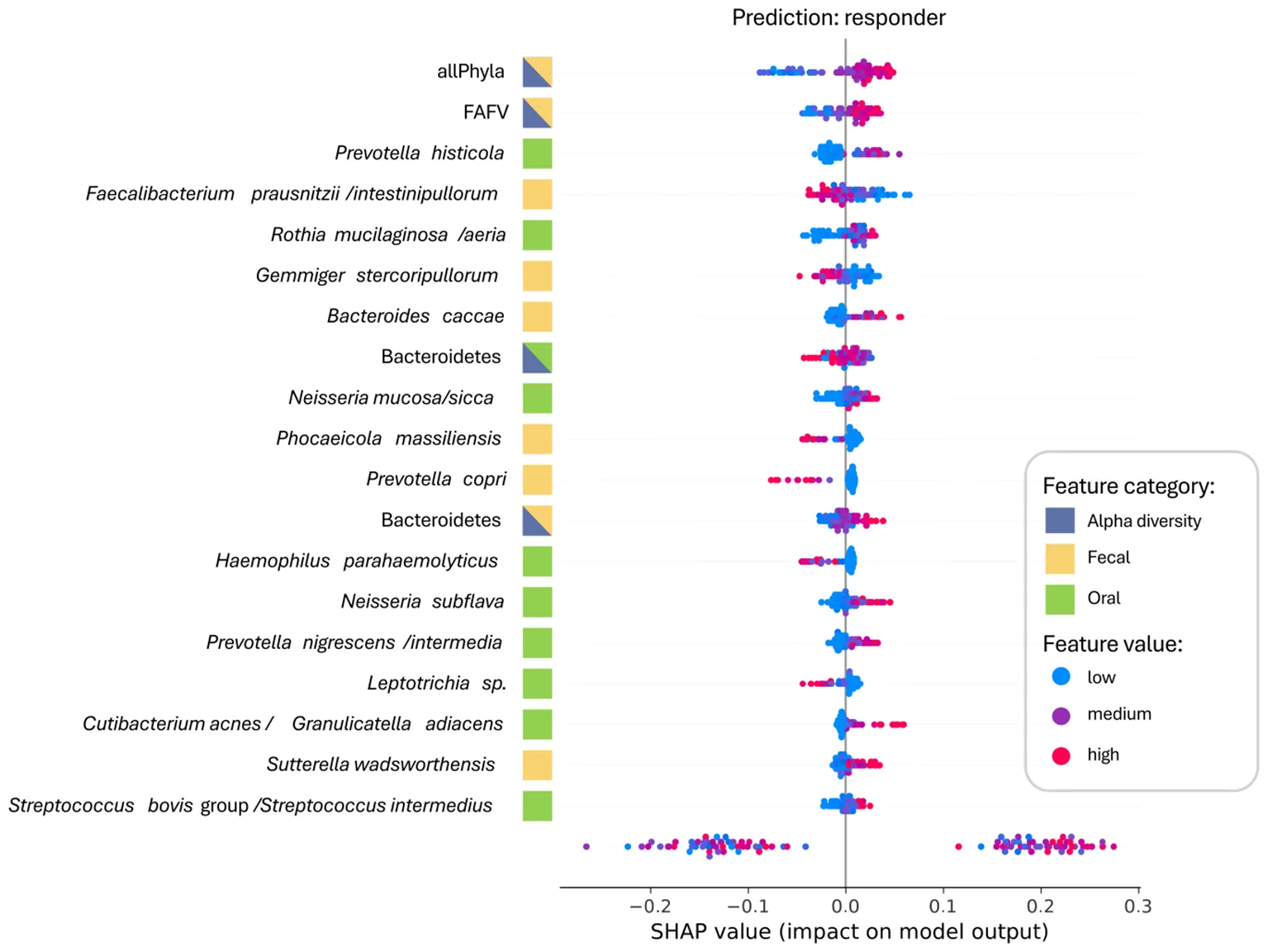
Most important features and impact on Random forest output. The x-axis includes shapley additive explanations (SHAP) values, if positive, the impact on the prediction (response) has a positive association. If negative, the value is negatively associated with the prediction. The y-axis includes the most important features sorted by feature importance and colored by category. The color of the dots indicates whether the feature has a high or low value (for prediction = ‘non-responder’ the x-axis is inverted).

In oral samples, high abundances of *Prevotella histicola* and *Rothia mucilaginosa/aeria* were associated with responders. In fecal samples, high abundances of *Bacteroides caccae* were associated with a good treatment response. Contrarily, a low abundance of *Faecalibacterium praunitzii/intestinipullorum* was associated with responders.

##### 3.4.2.3 Adaptative group regularized ridge regression

Since the results from the previous model were suboptimal for prediction purposes due to restrictions in sample size, a different method was introduced by applying the AGRR model. To test this notion, three models were compared using a Receiver Operating Characteristic (ROC) plot: ridge (standard ridge regression), GRidge (ridge regression using phylum and sample origin as co data), GRidge top 25 (selected features based on the group-weighted elastic net). The best performing model was GRidge top25 (AUC:0.642), followed by GRidge (AUC:0.609) and ridge (AUC:0.595) (**Supplementary Figure 5**).

The 25 features extracted from the elastic net included alpha diversity and relative abundances from all three phyla and species from both for fecal and oral samples (**Table 3**). Some of the parameters with the most influence were also part of the top features in the Random Forest model. These were alpha diversity overall (fecal), alpha diversity Bacteroidetes (fecal), *Bacteroides caccae* (fecal), *Neisseria mucosa/sicca* (oral) and *Prevotella copri* (fecal).

**Table 3:** GRidge top 25 features based on the group-weighted elastic net.

Species or alpha diversity	Category	Sample origin	β-value
FAFV	Alpha diversity	Fecal	0,090
allPhyla	Alpha diversity	Fecal	0,087
*Intestinibacter bacterium*	FAFV	Fecal	0,081
*Prevotella copri*	Bacteroidetes	Fecal	-0,078
Bacteroidetes	Alpha diversity	Fecal	0,077
*Faecalibacterium prausnitzii intestinipullorum*	FAFV	Fecal	-0,075
*Haemophilus parahaemolyticus*	Proteobacteria	Oral	-0,075
*Unknown FAFV*	FAFV	Oral	-0,069
*Phocaeicola massiliensis*	Bacteroidetes	Fecal	-0,069
*Anaerotruncus excrementipullorum*	FAFV	Fecal	-0,068
*Neisseria mucosa sicca*	Proteobacteria	Oral	0,067
*Barnesiella merdipullorum*	Bacteroidetes	Fecal	0,063
*Salmonella enterica*	Proteobacteria	Fecal	0,062
*Rothia mucilaginosa aeria*	FAFV	Fecal	-0,062
*Veillonella dispar*	FAFV	Oral	-0,061
FAFV	Alpha diversity	Oral	0,059
*Streptococcus cristatus*	FAFV	Fecal	0,058
*Lactobacillus iners*	FAFV	Fecal	0,058
*Sutterella megalosphaeroides*	FAFV	Fecal	0,058
*Streptococcus bovis group/Streptococcus intermedius*	FAFV	Oral	0,057
*Caproicibacterium lactatifermentans*	Proteobacteria	Fecal	-0,055
*Bacteroides caccae*	Bacteroidetes	Fecal	0,054
*Clostridium innocuum*	FAFV	Fecal	-0,052
*Streptococcus cristatus criceti*	FAFV	Oral	-0,051
*Bacteroides cellulosilyticus*	Bacteroidetes	Fecal	-0,048

β-value (or penalties) indicates the positive or negative contribution for the target label prediction. If positive, the impact on the prediction (response) has a positive association. If negative, the value is negatively associated with the prediction. FAFV, Firmicutes, Actinobacteria, Fusobacteria, and Verrucomicrobia.

The accuracy of the ridge model was 58.5% while the GRidge model was 59.5% (**Supplementary Table 5**). Sensitivity, specificity, PPV and NPV were also greater for GRidge. However, the small dataset size resulted in great confidence intervals which impaired the statistical impact of these results.

### 3.5 Association between immunological and microbiological profile

All immune cell populations were tested for association with each microbiological measure using LMM. After correction for multiple testing, no significant associations were found between immune cell populations and microbiological measures. Also, the interaction with response status was not significant for any comparison.

### 3.6 Food intake and correlation with microbiological measures

Food diary data were assessed after all visits to analyze changes in dietary patterns in relation to microbiota composition and treatment response between time points. Two participants did not submit any dietary diary data. A total of 53 participants (65%) completed all four 7-day diaries and 26 participants (32%) missed at least one diary time point.

#### 3.6.1 Fiber and fermented food intake

The average dietary fiber intake was suboptimal in the majority of participants, based on the World Health Organization’s recommendation of at least 25 grams/day (44). A total of 57% of participants were found to be consuming between 10 and 17 grams of fiber per day, while 16% consumed less than 10 grams per day. Only 18% were found to have a daily intake ranging from 17 to 20 grams, and a mere 9% exceeded 20 grams per day (**Figure 6A**). The fermented food intake was also quite low, with only 15% of participants having an intake of 1–2 portions/day (**Figure 6B**).

**Figure 6:**
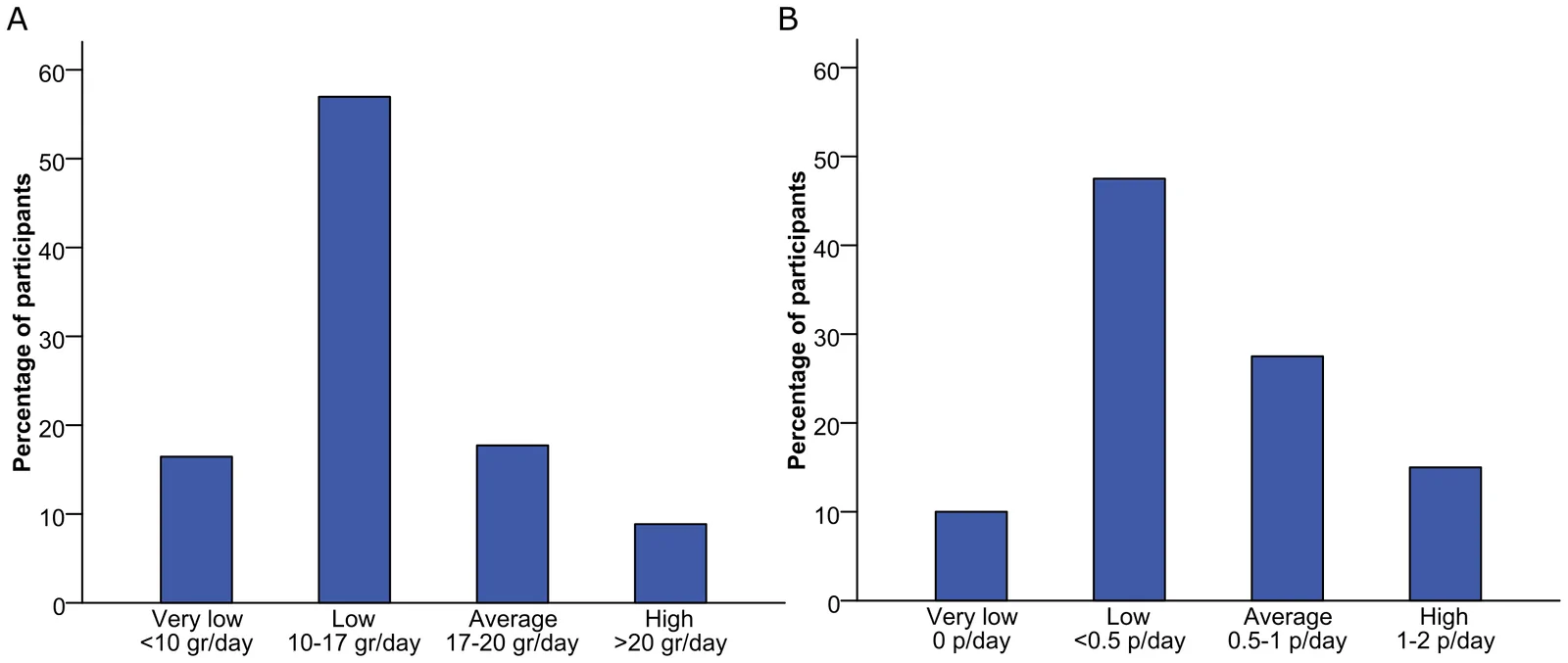
Distribution of daily dietary fiber and fermented food intake among participants. **(A)** Fiber intake. **(B)** Probiotic intake.

There was no significant change of the fiber and fermented food intake between time points in both responders and non-responders. Also, the average fiber and fermented food intake did not differ significantly between responders and non-responders (**Supplementary Figure 6**).

#### 3.6.2 Correlations between dietary habits and microbiological measures

To assess if dietary habits substantially affect the microbiota composition, correlation analyses were performed. No significant correlations were found between the average fiber intake per day and alpha diversity measures, relative abundance of phyla and F/B ratio in both fecal and oral samples. Also, no significant correlations were found between fermented food intake and alpha diversity measures, relative abundance of phyla and F/B ratio in both sample types (**Supplementary Table 6**).

## 4 Discussion

This study aimed to better understand the interaction between the gut-brain axis and CladT to improve treatment response prediction, by investigating changes in gut and oral microbiota and immunological profiles during treatment and evaluating microbiota as a predictor of two-year treatment response. Immune cell proportions did not differ between responders and non-responders. Network analysis revealed non-significant differences between responders and non-responders, with more positive correlations between immune cell clusters within the same parent cluster and a stronger negative correlation between CD8^+^ T cells and myeloid cells in responders. Microbiota did not differ significantly between groups, but prediction models identified a higher fecal alpha diversity (overall and Bacteroidetes), a high relative abundance of the species *Bacteroides caccae* in fecal samples and *Neisseria mucosa/sicca* in oral samples to be associated with a positive treatment response for CladT, while a high abundance of *Prevotella copri* in fecal samples was associated with a negative treatment response to CladT.

Immune cell reconstitution following CladT does not differ between responders and non-responders, as described before (45). Reconstitution was characterized by a depletion of naïve CD4^+^ and CD8^+^ T cells until M12, with recovery of only CD8^+^ T cells at M24, reflecting higher homeostatic proliferation and faster repopulation of naïve CD8^+^ T cells (46, 47). CD4^+^ T_CM_ cells were depleted at M3, and only partially recovered after the first year but not after the second year, suggesting a cumulative effect and more efficient ablation after two treatment cycles. Since CD4^+^ T_CM_ cells are clonally expanded antigen-primed cells that can rapidly expand and differentiate into effector memory cells upon antigen re-encounter, depleting these cells in MS is likely beneficial as it decreases the potential differentiation of autoreactive T cells (48). We found some fluctuations in both CD4^+^ and CD8^+^ T_EMRA_ populations, which has not been described before in relation to CladT, possibly due to their slow turnover limiting CladT’s effect (49). B cell proportions strongly decreased at M3. While most B cell subsets recovered by M24, memory B cells remained depleted. This can be explained by the slow repopulation of memory B cells compared to other B cells; where other B cells directly differentiate from bone marrow progenitors, memory B cells require germinal center reactivation, next to the fact that those germinal centers are also affected by CladT (11, 50). As B cell depletion is associated with high levels of disease control in both relapsing and progressive MS, this slow recovery of memory B cells is likely central to CladT’s effectiveness (50). Monocytes showed a slight initial increased followed by a gradual decrease, consistent with a low deoxycytidine kinase:5’ nucleotidase (DCK:5-NT) ratio and rapid regeneration, suggesting they are less affected than other immune cells (51, 52). Our findings align with previous literature on immunological changes after CladT (7, 8).

Network analysis showed that differences in immune cell correlation structure between responders and non-responders were most pronounced at 3 months after treatment. However, this difference did not reach statistical significance after correction (adjusted *p* = 0.3), though the nominal difference (*p* = 0.08) may point to a trend toward more coordinated immune reorganization in responders after CladT, warranting further investigation in larger cohorts. Positive correlations were predominantly within the same parent cluster, reflecting linked recovery of related subsets, such as specific CD4+ T cell clusters with their CD8+ counterpart. Negative correlations were observed primarily between monocytes and CD8^+^ T cell clusters, consistent with CladT exerting a stronger impact on T cells than monocytes (51). This correlation is stronger in responders and suggests better maintenance of immune homeostasis by preserving a more balanced interplay between lymphoid and myeloid compartments, potentially inhibiting autoreactive T cells.

Microbiological analysis revealed no significant differences in alpha diversity between time points between responders to non-responders, consistent with literature suggesting that alpha diversity is not a reliable biomarker across MS stages (53). This can be explained by most studies assessing total alpha diversity without dissecting it by phylum, potentially masking opposing richness changes across phyla, which may contribute to a state of dysbiosis. A higher overall fecal alpha diversity, and particularly of the Bacteroidetes phylum, was associated with CladT responders and raked among the strongest predictors in our models, reinforcing their key contribution. Often, the differences in alpha diversity described are tightly associated with diet; however, we found no further associations between microbiota richness and food intake.

‘Smoke’ status and the ‘Bristol stool scale’ indicated compositional changes in specific phyla. Smokers exhibited oral dysbiosis with an increase in the Proteobacteria phylum and a decrease in Bacteroidetes, a pattern not previously reported in healthy individuals but consistent with Li et al.’s findings in individuals with MS (54). Regarding fecal diversity, our research revealed a significant increase in Bacteroidetes in participants exhibiting a lower Bristol stool scale, likely reflecting greater microbiota richness associated with slower gastrointestinal transit (indicated by a low Bristol stool scale) (55).

Although our machine learning models only reached a poor discrimination (AUC <0.7) between responders and non-responders, we found several microbiological measure to be consistently associated with response status across both the random forest model and the AGRR (56). These parameters were the fecal alpha diversity (overall and Bacteroidetes phylum), the oral *Neisseria mucosa/sicca* and the fecal species *Bacteroides caccae* and *Prevotella copri.* No prior data exists on a relation between *Neisseria mucosa/sicca* and MS response status. A lower abundance of Bacteroidetes species in the gut microbiota has been established as an indicator of MS over healthy controls (57). This finding is connected to the metabolism of SCFA’s by *Bacteroides* sp. and its anti-inflammatory properties (58, 59). This is in line with our findings that high *Bacteroides caccae* abundance and high Bacteroidetes richness, are associated with a positive response status. *Prevotella copri* was significantly increased in non-responders, in line with a recent study about the oral dysbiosis of RRMS compared to a healthy cohort which found increased levels of the *Prevotella* genus in MS (60). In contrast, Li, et al. found potential beneficial microbes in the oral microbiota, including *Prevotella* species, illustrating the common inconsistency across microbiota studies regarding individual species (54). These inconsistencies underscore the importance of focusing on microbial functionality and community richness rather than individual species, supported by a comparable study achieving better predictive performance (0.76) using oral microbiota metabolites rather than species abundance (60, 61). This highlights an important limitation of our approach; as IS-pro provides only partial species-level resolution, it cannot provide functional inference, measurement of microbial metabolic activity, or identification of microbial genes and pathways, in contrast to shotgun metagenomic sequencing. Metabolic activity measurements in bacterial communities are largely validated *in vitro* and are difficult to apply *in vivo* (62). It would require radiolabeled metabolite tracing and repeated, invasive sampling, which is difficult to reconcile within the first months of treatment to predict response status timely. IS-pro therefore offers a more rapid and less invasive alternative suited to our research question.

No association between immunology and microbiology measures was found, possibly due to the complexity of their interaction. For example, we only investigated the proportion of immune cells and abundance of phyla and species, while the interaction might be caused by specific metabolites of some species or cytokines produced by several immune cells.

Dietary analysis showed no significant differences in fiber and fermented food intake between time points or between responders and non-responders. This suggests that the changes between time points we found in microbiota composition were not confounded by dietary factors. Also, we did not find any significant correlation between fiber and fermented food intake and the microbiota composition. Notably, both responders and non-responders showed consistently low fiber and fermented food intake, which may explain the absent diet-microbiota correlation and highlights the need for targeted dietary interventions to better understand diet’s role in MS management.

Strengths of this study are the longitudinal design of the study and the combination of both immunological and microbiological data, with special attention to the effect of dietary habits on the microbiota. Limitations include the use of IS-pro rather than shotgun metagenomic sequencing as discussed above. Additionally, the use of different prior IMT without washout, potentially affecting baseline findings. The absence of a re-baseline MRI risked misclassifying some participants as non-responders; correcting for this by analyzing response status for the second treatment year resulted in only 20% non-responders, which limited statistical power. Furthermore, although sample size estimations were based on prior publications, they did not account for the fact that microbiota samples tend to remain relatively stable over time (30). Because microbiota signatures are highly individual-specific, this stability can be exploited by machine learning algorithms, creating a risk of data leakage. To address this concern, we reduced our dataset and incorporated co-data. Additionally, immunological data were expressed as percentages of total cell populations rather than absolute cell counts, which may have limited our ability to detect and interpret changes in overall cell numbers. Finally, immunological analysis was performed in different batches with two different CyTOF systems, resulting in batch variability that was corrected through manual per-sample gating.

## 5 Conclusions

To conclude, we found a trend toward more coordinated immune reorganization in CladT responders, and identified fecal alpha diversity and Bacteroides caccae and Prevotella copri as associated with treatment response. Unfortunately, we could not develop a reliable model to predict treatment response before or shortly after the start of CladT. Our results warrant further investigation in larger cohorts.

## Data Availability

The datasets presented in this study can be found in online repositories. The names of the repository/repositories and accession number(s) can be found below: https://lifesciences.datastations.nl/dataset.xhtml?persistentId=doi:10.17026/LS/OARFZH, 10.17026.

## Group member of BIA study group

CMJ van Ansenwoude, MSc, Department of Molecular Cell Biology and Immunology, MS Center Amsterdam, Amsterdam Neuroscience, Amsterdam UMC Location Vrije Universiteit Amsterdam, Amsterdam, the Netherlands; J de Beukelaar, MD, PhD, Department of Neurology, Albert Schweitzer Hospital, Dordrecht, the Netherlands; ML Boon, MSc, inBiome, Amsterdam, the Netherlands; AE Budding, MD, PhD, inBiome, Amsterdam, the Netherlands; J Fermont, MD, Department of Neurology, Amphia Hospital, Breda, the Netherlands; STFM Frequin, MD, PhD, Department of Neurology, St Antonius Hospital, Nieuwegein, the Netherlands; OHH Gerlach, MD, PhD, Department of Neurology, Zuyderland Medical Center, Sittard-Geleen, the Netherlands, and School for Mental Health and Neuroscience, Maastricht University, Maastricht, the Netherlands; J Killestein, MD, PhD, MS Center Amsterdam, Department of Neurology, Amsterdam Neuroscience, Amsterdam UMC Location Vrije Universiteit Amsterdam, Amsterdam, the Netherlands; G Laureys, MD, PhD, Department of Neurology, University Hospital Ghent, Ghent, Belgium; M Miguelez, MSc, inBiome, Amsterdam, the Netherlands; BI Lissenberg-Witte, MSc, PhD, Department of Epidemiology and Data Sciences, Amsterdam UMC, Vrije Universiteit Amsterdam, Amsterdam, the Netherlands, and Department of Data Science and Biostatistics, Julius Center for Health Sciences and Primary Care, Universitair Medisch Centrum Utrecht, Utrecht University, Utrecht, the Netherlands; MJA Koel-Simmelink, MSc, PhD, Neurochemistry Laboratory, Department of Laboratory Medicine, Amsterdam Neuroscience, Neuroinflammation, Amsterdam UMC,Vrije Universiteit Amsterdam, Amsterdam, the Netherlands; J van Pamelen, MD, Department of Neurology, Elizabeth-TweeSteden Hospital, Tilburg, the Netherlands, and Bravis Hospital, Bergen op Zoom, the Netherlands, and MS Center Amsterdam, Neuroscience, Amsterdam UMC Location Vrije Universiteit Amsterdam, Amsterdam, the Netherlands; L van Olst, MSc, PhD, Department of Molecular Cell Biology and Immunology, MS Center Amsterdam, Amsterdam Neuroscience, Amsterdam UMC Location Vrije Universiteit Amsterdam, Amsterdam, the Netherlands, and Department of Biomedical Sciences, University of Groningen, University Medical Center Groningen, the Netherlands; BW van Oosten, MD, PhD, MS Center Amsterdam, Department of Neurology, Amsterdam Neuroscience, Amsterdam UMC Location Vrije Universiteit Amsterdam, Amsterdam, the Netherlands; SMA van der Pol, BSc, Department of Molecular Cell Biology and Immunology, MS Center Amsterdam, Amsterdam Neuroscience, Amsterdam UMC Location Vrije Universiteit Amsterdam, Amsterdam, the Netherlands; C Rodriguez-Mogeda, MSc, PhD, Department of Molecular Cell Biology and Immunology, MS Center Amsterdam, Amsterdam Neuroscience, Amsterdam UMC Location Vrije Universiteit Amsterdam, Amsterdam, the Netherlands; NM de Roos, MSc, PhD, Division of Human Nutrition and Health, Wageningen University and Research, Wageningen, the Netherlands; T van Seeters, MD, PhD, Department of Radiology, Elisabeth-TweeSteden Hospital, Tilburg, the Netherlands; L Spijkerbosch, MSc, Division of Human Nutrition and Health, Wageningen University and Research, Wageningen, the Netherlands; CE Teunissen, MSc, PhD, Neurochemistry Laboratory, Department of Laboratory Medicine, Amsterdam Neuroscience, Neuroinflammation, Amsterdam UMC, Vrije Universiteit Amsterdam, Amsterdam, the Netherlands; LH Visser, MD, PhD, Department of Neurology, Elizabeth-TweeSteden Hospital, Tilburg, the Netherlands; J van Vliet, MD, PhD, Department of Neurology, Jeroen Bosch Hospital, ‘s-Hertogenbosch, the Netherlands; and HE de Vries, MSc, PhD, Department of Molecular Cell Biology and Immunology, MS Center Amsterdam, Amsterdam Neuroscience, Amsterdam UMC Location Vrije Universiteit Amsterdam, Amsterdam, the Netherlands.

## Glossary

AGRR: adaptive group-regularized logistic ridge regression
AUC: area under the curve
BIA: brain-immune-intestine axis
BY: Benjamini-Yekutieli
CAS+: cell acquisition solution plus
cDCs: conventional dendritic cells
CSB: cell staining buffer
CladT: cladribine tablets
CyTOF: mass cytometry by time-of-flight
DCK:5-NT ratio: deoxycytidine kinase:5’ nucleotidase ratio
EAE: experimental autoimmune encephalomyelitis
EDSS: expanded disability status scale
FCS: fetal calf serum
FAFV: Firmicutes, Actinobacteria, Fusobacteria, and Verrucomicrobia
F/B ratio: Firmicutes/Bacteroidetes ratio
T cells: gamma-delta T cells
IMT: immunomodulatory treatment
IQR: interquartile range
IS: interspace
IS-pro: interspace-profiling
LMM: linear mixed model
MRI: magnetic resonance imaging
MS: multiple sclerosis
NEDA: no evidence of disease activity
NK cells: natural killer cells
NKT-like cells: NK T-cell like cells
NPV: negative predictive value
PBMC: peripheral blood mononuclear cell
PCoA: Principal Coordinates Analysis
pDCs: plasmacytoid dendritic cells
PERMANOVA: Permutational Multivariate Analysis of Variance
PPV: positive predictive value
pwMS: people with multiple sclerosis
ROC: receiver operating characteristic
RRMS: relapsing-remitting multiple sclerosis
SCFA: short-chain fatty acids
SHAP: shapley additive explanations
TCM: central memory T cells
TEM: effector memory cells
TEMRA: effector memory cells re-expressing CD45RA T cells
UMAP: uniform manifold approximation and projection
