# Supplementary material for: A two year clinical, immunological and gut-brain-axis prospective study to predict treatment response to cladribine tablets in relapsing-remitting multiple sclerosis
Source: Front Immunol. 2026 Oct 19;17:1955097. doi: 10.3389/fimmu.2026.1955097 (PMC13733286; doi:10.3389/fimmu.2026.1955097)
Supplement: Supplementary file 1 [file Table1.docx]

# Supplementary Table 1 Response status

|  | **Number of patients (%)** | |
| --- | --- | --- |
|  | **Overall** | **Year 2** |
| **Non-responder** | 39 (53%) | 14 (20%) |
| *Relapse^a^* | 5 (13%) | 0 (0%) |
| *Radiological disease activity^a^* | 27 (69%) | 10 (71%) |
| *Disability progression^a^* | 9 (23%) | 4 (29%) |
| *Only disease activity in first treatment year* | 24 (62%) | Not applicable |
| **Responder** | 35 (47%) | 57 (80%) |

Overall: response status for year 1 and year 2. Year 2: response status for year 2.
Overall: 7 patients were missing data on response status (4 because of lost to follow-up, 2 because of missing EDSS, and 1 because of missing relapse status). Two other patients were missing an EDSS, but their response status could be determined based on the parameters that were available.
Year 2: 10 patients were missing data on response status (as above, and 1 because of missing EDSS and 2 because of switch to another IMT)
^a^ Percentages don’t add up to 100 because of overlap of categories.

# Supplementary Table 2 Baseline characteristics split for response status

| Characteristics | Responders (n=35) | Non-responders  (n=39) | P-value |
| --- | --- | --- | --- |
| **Age, years, median (IQR)** | 47 (31.0-53.0) | 40 (31.0-45.0) | 0.08 |
| **Female, n (%)** | 24 (68.6) | 28 (71.8) | 0.80 |
| **Body mass index, median (IQR)** | 26.7 (23.9-29.1) | 23.3 (20.7-28.3) | **0.04** |
| **Disease duration, years, median (IQR)** | 8 (1.0-16.0) | 5.5 (2.0-10.0) | 0.24 |
| **Documented relapses in past 2 years, median (IQR)** | 1 (0-1) | 0 (0-1) | 0.13 |
| **IMT used in last 6 months, n (%)**  **(Peg-)Interferon β**  **Glatiramer acetate**  **Dimethylfumarate**  **Teriflunomide**  **Fingolimod**  **Natalizumab**  **Ocrelizumab**  **Other**  **None** | 3 (8.6)  4 (11.4)  8 (22.9)  10 (28.6)  3 (8.6)  1 (2.9)  0 (0)  1 (2.9)  5 (14.3) | 3 (7.7)  3 (7.7)  13 (33.4)  8 (20.5)  5 (12.9)  1 (2.6)  0 (0)  2 (5.1)  4 (10.3) | 0.87 |
| **Reason for switch to cladribine tablets, n (%)**  **Treatment naïve**  **Disease activity  Adverse events** | 5 (14.3)  26 (74.3)  4 (11.5) | 4 (10.3)  25 (64.1)  10 (25.7) | 0.20 |
| **Use of systemic corticosteroids in previous month, n (%)** | 1 (2.9) | 3 (7.7) | 0.62 |
| **Use of antibiotics in previous month, n (%)** | 2 (5.7) | 6 (15.4) | 0.27 |
| **Smoking, n (%)**  **Yes**  **Past**  **Never** | 5 (14.3)  13 (37.1)  17 (48.6) | 4 (10.3)  14 (35.9)  21 (53.8) |  |
| **EDSS at enrollment, median (IQR)** | 3.0 (1.5-3.5) | 2.0 (1.5-3.1) | 0.22 |

7 patients were missing data on response status. Significant	-values are represented in bold.

Abbreviations: EDSS, expanded disability status scale; IMT, immunomodulatory treatment; IQR, interquartile range.

# Supplementary Table 3 Linear mixed model results of immunological changes between time points in responders and non-responders and between groups

| **Immune cell** | **Response status** | ***P*-value**  **(between groups)** | **D0-M3** | **D0-M12** | **D0-M24** | **M3-M12** | **M3-M24** | **M12-M24** |
| --- | --- | --- | --- | --- | --- | --- | --- | --- |
| CD4^+^ naïve T cells | R | 0.808 | **0.001** | **0.005** | **0.007** | 1 | 1 | 1 |
|  | NR |  | **0.004** | **0.010** | **0.004** | 1 | 1 | 1 |
| CD4^+^ T_EM_ | R | 0.943 | 1 | 1 | 1 | 1 | 1 | 1 |
|  | NR |  | 1 | 1 | 1 | 1 | 1 | 1 |
| CD4^+^ T_CM_ | R | 0.637 | **0.042** | 0.315 | **0.005** | 0.941 | 0.941 | 0.315 |
|  | NR |  | 0.249 | 1 | 0.249 | 0.561 | 1 | 0.598 |
| CD4^+^ T_EMRA_ | R | 0.283 | 0.776 | 1 | **0.015** | 0.832 | 0.213 | **0.017** |
|  | NR |  | 0.379 | 0.379 | 0.350 | 1 | 1 | 1 |
| γδ T cells | R | 0.610 | 0.128 | 0.150 | 0.057 | 1 | 1 | 1 |
|  | NR |  | 0.279 | 1 | 0.708 | 0.708 | 1 | 1 |
| CD4^-^ CD8^-^ T cells | R | 0.729 | 1 | 1 | 1 | 1 | 1 | 1 |
|  | NR |  | 0.713 | 1 | 0.319 | 0.319 | **0.045** | 0.713 |
| CD8^+^ naïve T cells | R | 0.846 | **<0.001** | **0.008** | 0.614 | 0.614 | **0.008** | 0.159 |
|  | NR |  | **<0.001** | **0.022** | 0.497 | 0.497 | **0.030** | 0.459 |
| CD8^+^ T_EM_ | R | 0.341 | 0.790 | 1 | 0.790 | 0.917 | 1 | 0.790 |
|  | NR |  | 0.234 | 1 | 1 | 0.234 | 0.234 | 1 |
| CD8^+^ T_CM_ | R | 0.921 | 0.913 | 1 | 0.913 | 0.913 | 1 | 1 |
|  | NR |  | 1 | 1 | 1 | 0.970 | 1 | 1 |
| CD8^+^ T_EMRA_ | R | 0.280 | **0.033** | 1 | **0.033** | **0.050** | 1 | **0.050** |
|  | NR |  | **0.008** | 1 | 1 | **0.037** | 0.102 | 1 |
| CD4^+^ CD8^+^ T cells | R | 0.812 | 1 | 0.949 | 0.542 | 1 | 1 | 1 |
|  | NR |  | 0.457 | 0.128 | 0.457 | 1 | 1 | 1 |
| NKT-like cells | R | 0.854 | 0.537 | 0.537 | 1 | 1 | 1 | 1 |
|  | NR |  | **0.021** | 0.284 | 0.284 | 0.914 | 0.919 | 1 |
| CD56^bright^ NK cells | R | 0.578 | 1 | 1 | 0.480 | 1 | 0.880 | 0.880 |
|  | NR |  | 1 | 1 | 1 | 1 | 1 | 1 |
| CD56^dim^ CD57^-^ NK cells | R | 0.908 | 1 | 1 | 1 | 1 | 1 | 1 |
|  | NR |  | 1 | 1 | 0.173 | 1 | 0.173 | 0.173 |
| CD56^dim^ CD57^+^ NK cells | R | 0.669 | 0.074 | 1 | 1 | 0.074 | 0.074 | 1 |
|  | NR |  | 0.095 | 1 | 0.859 | 0.095 | 0.849 | 0.849 |
| CD27^-^ B cells | R | 0.954 | **0.005** | 0.151 | **0.005** | **<0.001** | **<0.001** | 0.503 |
|  | NR |  | **<0.001** | **0.020** | **<0.001** | **<0.001** | **<0.001** | 0.537 |
| Memory B cells | R | 0.646 | **<0.001** | **<0.001** | **<0.001** | 0.054 | 0.252 | 1 |
|  | NR |  | **<0.001** | **<0.001** | **<0.001** | **0.003** | 0.225 | 0.459 |
| Monocytes | R | 0.546 | **<0.001** | 0.148 | 0.757 | 0.148 | **0.025** | 0.812 |
|  | NR |  | **0.016** | 0.914 | 0.914 | 0.177 | 0.240 | 1 |
| cDCs | R | 0.571 | 0.179 | 0.926 | 0.978 | 0.960 | 0.926 | 1 |
|  | NR |  | 0.893 | 1 | 1 | 1 | 0.550 | 0.893 |
| pDCs | R | 0.238 | 0.360 | 1 | 1 | 0.557 | 0.617 | 1 |
|  | NR |  | 0.324 | 1 | 0.151 | 0.191 | **0.002** | 0.191 |
| Undefined | R | 0.450 | 1 | 1 | 1 | 1 | 0.661 | 0.661 |
|  | NR |  | 1 | 0.525 | 0.259 | 0.350 | 0.221 | 1 |

Table displays Benjamini-Yekutieli corrected *P*-values. *P*-value (between groups) displays the *p*-value of the LMM with overall response status as interaction term. The other columns display the p-value of changes over time in either responders (R) or non-responders (NR). P-values in bold are statistical significant after correction for multiple testing.

Abbreviations: NKT-like cells, natural killer-T like cells; γδ T cells, gamma-delta T cells; T_EMRA_, effector memory cells re-expressing CD45RA; T_CM_, central memory T cells; T_EM_, effector memory T cells; NK cells, natural killer cells; pDCs, plasmacytoid dendritic cells; cDCs, conventional dendritic cells

# Supplementary Table 4 PERMANOVA performance on the average profiles per patient from both sample origins

| **Sample origin** | **R^2^** | **F** | ***p*-value** |
| --- | --- | --- | --- |
| Fecal | 0.0125 | 0.91 | 0.579 |
| Oral | 0.0171 | 1.25 | 0.127 |

R^2^ measures the exact proportion of total multivariate variation explained by the response status. F represents the pseudo-F statistic, which measures the ratio of between-group variation to within-group variation based on a distance matrix. *P*-value states whether the group centroids differ in the multivariate space, and therefore whether there is a difference between groups.

# Supplementary Table 5 Ridge regression performance metrics

|  | **GRridge** | | **Top25 GRidge** | |
| --- | --- | --- | --- | --- |
| **Statistic** | **Value** | **95% CI** | **Value** | **95% CI** |
| Sensitivity (%) | 54.8 | 38.7 - 70.1 | 56.4 | 39.6 - 72.2 |
| Specificity (%) | 62.5 | 43.7 - 78.9 | 62.9 | 44.9 - 78.5 |
| Positive Predictive Value (PPV) | 61.3 | 48.3 - 72.8 | 62.2 | 49.7- 73.3 |
| Negative Predictive Value (NPV) | 56.1 | 45.4 - 66.2 | 57.1 | 46.2- 67.4 |
| Accuracy (%) | 58.5 | 46.4 - 69.8 | 59.5 | 47.5 - 70.8 |

# Supplementary Table 6 Correlation between fiber and probiotic intake and microbiota composition

| **Microbiota measure** | **Intake** | **Pearson correlation** | ***P-*value** | **Corrected *p-value*** |
| --- | --- | --- | --- | --- |
| F - Alpha diversity overall | Fiber | 0.006 | 0.959 | 0.959 |
| F - Alpha diversity FAFV |  | 0.115 | 0.314 | 0.628 |
| F - Alpha diversity Bacteroidetes |  | -0.085 | 0.458 | 0.733 |
| F - Alpha diversity Proteobacteria |  | 0.131 | 0.249 | 0.628 |
| F - Relative abundance FAFV |  | -0.276 | **0.014** | 0.112 |
| F - Relative abundance Bacteroidetes |  | 0.305 | **0.006** | 0.096 |
| F - Relative abundance Proteobacteria |  | -.009 | 0.940 | 0.959 |
| F - F/B ratio |  | -0.234 | 0.038 | 0.203 |
| O - Alpha diversity overall | Fiber | -0.060 | 0.599 | 0.744 |
| O - Alpha diversity FAFV |  | -0.055 | 0.630 | 0.744 |
| O - Alpha diversity Bacteroidetes |  | -0.093 | 0.416 | 0.733 |
| O - Alpha diversity Proteobacteria |  | 0.052 | 0.651 | 0.744 |
| O - Relative abundance FAFV |  | 0.117 | 0.306 | 0.628 |
| O - Relative abundance Bacteroidetes |  | -0.182 | 0.108 | 0.432 |
| O - Relative abundance Proteobacteria |  | 0.060 | 0.602 | 0.744 |
| O - F/B ratio |  | 0.116 | 0.308 | 0.628 |
| F - Alpha diversity overall | Probiotic | -0.008 | 0.941 | 0.972 |
| F - Alpha diversity FAFV |  | -0.079 | 0.488 | 0.972 |
| F - Alpha diversity Bacteroidetes |  | -0.019 | 0.870 | 0.972 |
| F - Alpha diversity Proteobacteria |  | 0.163 | 0.150 | 0.800 |
| F - Relative abundance FAFV |  | 0.004 | 0.972 | 0.972 |
| F - Relative abundance Bacteroidetes |  | 0.215 | 0.056 | 0.448 |
| F - Relative abundance Proteobacteria |  | -0.228 | **0.042** | 0.448 |
| F - F/B ratio |  | -0.073 | 0.521 | 0.972 |
| O - Alpha diversity overall | Probiotic | -0.013 | 0.908 | 0.972 |
| O - Alpha diversity FAFV |  | -0.121 | 0.286 | 0.972 |
| O - Alpha diversity Bacteroidetes |  | 0.062 | 0.588 | 0.972 |
| O - Alpha diversity Proteobacteria |  | 0.088 | 0.437 | 0.972 |
| O - Relative abundance FAFV |  | 0.052 | 0.648 | 0.972 |
| O - Relative abundance Bacteroidetes |  | -0.080 | 0.481 | 0.972 |
| O - Relative abundance Proteobacteria |  | 0.030 | 0.794 | 0.972 |
| O - F/B ratio |  | 0.030 | 0.794 | 0.972 |

*P*-values in bold are significant. Abbreviations: F, fecal; O, oral

# Supplementary Figure 1 Gating strategy of immune cell clusters by mass cytometry by time-of-flight (CyTOF)

Live CD45^+^ cells were gated based on canonical lineage markers. Grey arrows show sequential gating steps, black arrows indicate the final gated cluster. Representative plots from one donor are displayed.
Abbreviations: NKT-like cells, natural killer-T like cells; γδ T cells, gamma-delta T cells; T_EMRA_, effector memory cells re-expressing CD45RA; T_CM_, central memory T cells; T_EM_, effector memory T cells; NK cells, natural killer cells; pDCs, plasmacytoid dendritic cells; cDCs, conventional dendritic cells

# Supplementary Figure 2 Immune cell composition alterations in non-responders of cladribine treatment

Barplots depict percentages of identified clusters out of the total CD45+ cells between time points in non-responders. Columns and error bars represent mean ± SD, each dot represents an individual donor. Changes in proportions were analyzed using a linear mixed-effects model adjusting for age and sex and including a random intercept for patient.
Abbreviations: NKT-like cells, natural killer-T like cells; γδ T cells, gamma-delta T cells; T_EMRA_, effector memory cells re-expressing CD45RA; T_CM_, central memory T cells; T_EM_, effector memory T cells; NK cells, natural killer cells; pDCs, plasmacytoid dendritic cells; cDCs, conventional dendritic cells

# Supplementary Figure 3 Changes of microbiological measures between time points in responders and non-responders (‘overall response status’)

Columns and error bars represent median and IQR, whisker represents range. Colored circles represent outliers (1.5 IQR below the first or above the third quartile) and colored asterixis represent extreme outliers (3 IQR below the first or above the third quartile). **A.** Alpha diversity. **B.** Relative abundance of phyla. **C.** Firmicutes/Bacteroidetes Ratio.
Abbreviations: bact, Bacteroidetes; FAFV, Firmicutes, Actinobacteria, Fusobacteria, and Verrucomicrobia; proteo, Proteobacteria.

# Supplementary Figure 4 PCoA plots for responders and non-responders A B

PCoA plots based on cosine distance of fecal (A) and oral (B) microbiome composition. Each point represents an individual participant, colored by response status (responders, red; non-responders; blue). Ellipses represent 95% confidence intervals. Group separation was not significant by PERMANOVA (fecal: R^2^ = 0.01, *p* = 0.5, oral: R^2^ = 0.01, *p* = 0.1).

# Supplementary Figure 5 ROC plot AGRR models including all co-data

The x-axis displays the False positive rate (FPR), while the y-axis displays the True positive rate (TPR). The co-data used for this model includes the p-values obtained from the LMM, ‘Category’ (Alpha diversity, Bacteroides, FAFV and Proteobacteria) or ‘Sample origin’ (Fecal and Oral) of the parameters. The dashed red line represents a random classifier. The gray line represents the baseline ridge model. The solid black line represents the Grouped Ridge (GRidge), while the dashed one displays the model containing exclusively the top 25 most important features.

# Supplementary Figure 6 Changes between time points in fiber and probiotic intake compared between responders and non-responders

A: fiber intake. B: probiotic intake. Abbreviations: R, responders; NR, non-responders.
